# Various structural factors influenced early antiretroviral therapy initiation amongst HIV infected prisoners: a qualitative exploration in South Ethiopia

**DOI:** 10.1186/s12889-021-11499-w

**Published:** 2021-07-28

**Authors:** Terefe Gone Fuge, George Tsourtos, Emma R. Miller

**Affiliations:** College of Medicine and Public Health, Flinders University, Adelaide, Australia

**Keywords:** Antiretroviral therapy, Initiation, Prisoners, South Ethiopia, Qualitative interviewing

## Abstract

**Background:**

Early initiation of antiretroviral therapy (ART) reduces the development of acquired immunodeficiency syndrome (AIDS), non-AIDS related comorbidities and mortality, and prevents transmission. However, the prevalence of delayed ART initiation amongst prisoners in sub-Saharan African countries is high and the contributing factors to this are relatively unknown.

**Methods:**

Qualitative interviewing was employed to understand the prisoners’ lived world with regard to initiating ART and associated barriers and facilitators in the South Ethiopian prison system. We interviewed seven (five male and two female) inmates living with HIV (ILWH) and eleven stakeholders who had a role in human immunodeficiency virus (HIV) care provision for incarcerated people. A phenomenological approach was used to analyse the interview data in which meaning attributed to the lived experiences of the participants was abstracted.

**Results:**

In this study, participants discussed both barriers to, and facilitators of, early ART initiation during incarceration. The barriers included a lack of access to voluntary counselling and testing (VCT) services, poor linkage to care due to insufficient health staff training, uncooperative prison security systems and loss of privacy regarding disclosure of HIV status. Insufficient health staff training and uncooperative prison security systems both contributed to a loss of patient privacy, ultimately resulting in treatment refusal. Although most participants described the importance of peer education and support for enhancing HIV testing and treatment programs amongst prisoners, there had been a decline in such interventions in the correctional facilities. Service providers suggested opportunities that a prison environment offers for identification and treatment of HIV infected individuals and implementation of peer education programs.

**Conclusions:**

Our study identified crucial barriers to and facilitators of early ART initiation amongst prisoners, a key HIV priority population group. Interventions that address the barriers while strengthening the facilitators may enhance a greater utilisation of ART.

**Supplementary Information:**

The online version contains supplementary material available at 10.1186/s12889-021-11499-w.

## Background

Incarceration rates have been increasing rapidly in sub-Saharan African (SSA) countries including Ethiopia [1, 2]. Socioeconomic and political circumstances such as unemployment, economic inequalities and widespread conflict (leading to an imprisonment of political opponents and related offenders) are important drivers of incarceration in the countries [3, 4]. In Ethiopia, there are currently 113,727 people in prison giving an imprisonment rate of 127 per 100,000 population, which is one of the highest rates in East Africa [2].

The prevalence of human immunodeficiency virus (HIV) in prison populations of SSA countries is much higher than in the general population [5–7], with prevalences up to 13 times higher reported in some countries [5]. In Ethiopia, the prevalence of HIV in prison populations is more than four times higher than in the general population [8]. While there have been reports of unprotected homosexual practices, including rape and sex bartering in the SSA prisons [9–13], pre-incarceration unprotected heterosexual intercourse is the main risk factor for HIV infection in prisoners [9, 11, 14, 15].

Early antiretroviral therapy (ART) initiation; i.e. commencing ART immediately after the infection occurs [16] or as World Health Organization (WHO) defines: initiating ART at higher CD4 count and/or lower clinical stages [17] not only reduces the development of acquired immunodeficiency syndrome (AIDS) and non-AIDS related comorbidities and mortality [18, 19], but also prevents transmission of HIV infection in the community by reducing viral concentration in people living with HIV (PLWH) [20, 21]. Imprisonment provides a unique opportunity to implement comprehensive HIV care programs including timely ART initiation, as PLWH who may otherwise not access health care can be readily reached. High treatment initiation rates have been reported in prison settings, although this is only where there exists an established structure for HIV care [22–24].

In many nations, however, inmates living with HIV (ILWH) often have delayed treatment initiation despite bearing the highest burden of infection and the potential for HIV transmission in the community upon release. Various structural, social and individual level factors have been reported to adversely influence ART initiation for prisoners. Identified structural factors include: a lack of standard HIV care in the prison system [25–29], lack of trust in health care providers [30, 31], loss of privacy [32], discriminatory treatment by prison staff and protracted processes involved in clinic visits [26–28, 32–34]. Social factors, such as stigmatisation by prison officers and fellow inmates, are also found to adversely influence treatment initiation for prisoners [32, 35]. Moreover, a lower likelihood of treatment initiation has been reported in prisoners who believe that ART is unsafe and ineffective in treating HIV [30, 35, 36].

Specific factors contributing to delayed ART initiation amongst prisoners in low- and middle-income countries are unclear. The few available studies that have investigated this issue [25–29] suggested that suboptimal HIV care in the prison system might have caused delay in ART initiation. The purpose of this study was therefore to explore barriers to, and facilitators of, early treatment initiation amongst HIV infected prisoners within the context of South Ethiopia.

## Methods

### Study setting

South Ethiopia, which is also administratively called Southern Nations, Nationalities and People’s Region (SNNPR), is home to 23 of 126 prisons present in Ethiopia [37]. According to SNNPR Prison Commission data [38], the annual imprisonment rate in the region was104 per 100,000 population as of March 2020, with a total prison population of 24,628.

Four SNNPR prisons and respective public health care facilities offering ART services for the prisoners were included in the current study. The prisons were selected based on the number of inmates they house and sociocultural diversity amongst their prisoner populations. The prisons are located in the central part of Ethiopia and accommodate people originating from diverse areas of the region and the country. This is believed to enhance the representativeness of information beyond the included prisons. The prisons had a daily average number of approximately 5500 inmates, with an average daily entry of 15 persons in each prison [38]. The prisons serve both male and female prisoners in separate units. The majority of prisoners were male, accounting for an average of 96% of the total prison population.

All four prisons had health clinics that were equipped with basic health care materials and health care staff, including psychologists. The prison health care staff mostly consisted of nurses, health officers and laboratory technicians who provided basic care, including first level diagnostic and treatment services for emergency cases and acute illnesses commonly encountered in Ethiopia such as malaria and tuberculosis. None of the clinics operated comprehensive ART services. However, they occasionally offered voluntary counselling and testing (VCT) services and referred identified HIV-infected inmates to nearby ART facilities for treatment initiation [39].

### Design

Qualitative in-depth interviewing using a phenomenological approach was undertaken to explore the lived experiences of HIV infected prisoners regarding ART initiation in the prison context. In-depth semi-structured interviews were also employed to explore the service providers’ experiential account of the existing HIV care provision strategy.

### Participants

Our aim was to purposively target ILWH and relevant service providers who met study eligibility criteria. ILWH participants were those who were 18 years or older and had initiated ART after prison entry. In addition, the prisoner participants were required to be fluent in Amharic language (a widely spoken language in Ethiopia) in order to maintain verbal fluency and clarity of ideas to the interviewer.

The service provider group included adults (≥18 years of age) who were members of prison health care staff, ART service providers, prison officers, and prison and health administrators. Participants of this group were selected based on their role in the process of HIV care provision. The prison health care staff were health professionals working within the prison health care system and had experiences of performing HIV test and linking HIV infected prisoners to care, whereas the ART service providers were those who were providing HIV treatment for incarcerated and non-incarcerated people at the selected public health care facilities. The prison officers were members of prison security who were often involved in the facilitation of prisoners’ HIV care accessing. The prison administrators were the higher officials of the prisons managing the overall administrative issues of the institutions including HIV care, whereas the health administrators were health agents in the respective Zonal Health Departments who were providing technical as well as material support for the prison health care system. All service provider participants had over 6 months’ working experience in their respective positions.

### Data collection

The interaction between the interviewer and participants was open-ended, in order to create sufficient room for reflections [40, 41], however an interview guide was used to preserve the focus of the discussions on issues and processes related to ART initiation in the prison context (see Additional file 1). The interview guide was constructed in relation to the literature review and the research question. The interview guide for prisoners asked questions related to physical and social environments promoting and hindering early ART initiation, and personal contexts regarding inmates’ understanding and perception towards ART. Service provider participants were asked to provide accounts of what they had noticed during their engagement with the provision of HIV care for incarcerated people.

Eighteen participants from the four selected prisons and respective supporting health care facilities participated in the in-depth interviews including: seven (five male and two female) prisoners, two prison health care staff, three ART service providers, two prison officers, two prison administrators and two health agents. The number of participants was determined based on theoretical saturation and a diversity of participants with regard to prison settings, role in the provision of care as well as range of experience [42].

Prisoners were interviewed in a private secured place by the principal researcher (TGF); either in a prison clinic or a room near to it. Due to security concerns in prison settings, prison health care staff guided the researcher to contact the prisoners in order to obtain consent for voluntary participation when they made their regular clinic visits. The principal researcher retrieved clinic appointments of all eligible prisoners from medical registers prior to the commencement of the interviews. This was believed to minimise a potential bias due to the involvement of the health staff in the participant selection process. In addition, the health staff played no role during consent and interviewing processes. Service provider participants were interviewed in their respective offices in private. Prisoner interviews took up to 60 min whereas that of the service provider took a maximum of 50 min. All interviews were audio recorded and field notes were taken on observations (tacit knowledge), comments, unclear ideas and emerging insights [40, 43]. The principal researcher initially transcribed the audio recorded interview data in Amharic language and then translated into English for analysis.

To ensure reliability and credibility of the interview data, the interview guide was initially piloted with individuals from the target population (two for prisoner participants and one for each category of service provider participants) at institutions other than the study sites. This allowed identification of elements that supported the objectives of the study, inclusion of relevant concepts that had not been considered previously and modification of those which were found to be incomprehensible to the participants. The pilot interviews also enabled the interviewer to explore unanticipated circumstances involved in the interviewing process within prison context [41]. Rapport was established with both prisoner and service provider participants through sharing of the interviewer’s experience in issues related to HIV care while retaining the distance essential to explore their views.

### Analysis

Analysis of data was conducted iteratively throughout the interviewing process. The analysis employed a phenomenological approach so as to contextualise abstract meanings attributed to the lived experiences of the participants regarding ART initiation in the prison system [44, 45]. Meaning was attached to a particular phenomenon by participants, but inferred using relevant antecedent theories [44].

Data were initially understood through repeated readings of transcripts and review of field notes for tacit information [40]. Themes were first drawn from the data through detection of shifts in meaning and comparison of emerging themes within and between transcripts [40, 45, 46]. There was consensus, after discussion and debate, on most of the themes identified from triangulating perspectives and interpretations between three analysts (a male postgraduate student (TGF), one female academic (ERM) and one male academic (GT)). The themes were coded and juxtaposed in a chronological order of events and conceptual relationships using NVivo12 qualitative data analysis software [47].

Interpretations were made by comparing emerging concepts horizontally within and between the different categories of participants (i.e. amongst prisoner participants and between prisoner and service provider participants), prison settings and phenomena, and vertically between the themes (emic categories) and theoretical concepts (etic categories) in terms of recurrence, patterns and relationships [40, 48].

Reflexivity was considered important for the analysis regarding the influence that the primary researcher (a male Ethiopian post graduate student who had no previous experience of imprisonment) may have had during his interactions with prisoner and service provider participants, and while analysing and interpreting the interview data. The researcher belonged to the same ethnic background and shared many of the same cultural practices from which most of the prisoner participants originated, which might have given him to some extent an insider role to access the culture and ask participants more meaningful questions [49, 50]. While the potential difference in socioeconomic and educational status between the researcher and prisoner participants might have impacted the trustworthiness of data, the researcher’s previous research experiences in the same settings [51] offered him an opportunity to understand the research context [49, 50]. The researcher constantly maintained a journal of the research process encompassing experiences, emotions and change in attitudes towards participants and how this could impact data [52]. Data were interpreted by triangulating the perspectives of two other researchers (non-Ethiopian male and female academics) which could potentially reduce bias due to the researcher’s view of prisoner participants in terms of accessing HIV care within the context of the study setting [50, 53]. Participants were provided with the summary of the results and asked to verify the accuracy of the results (member-checking) when data interpretation was completed [41, 50, 54]. Whereas prisoner participants were provided with the results through their prison’s postal address, an email address was used for service provider participants. All participants verified the accuracy and agreed with the results.

## Results

### Participant characteristics

Prisoner participants had a median age of 36.5 years. Most (five) of the prisoners reported elementary school (1-8th grade) as their highest educational attainment. Five inmates had been incarcerated for more than 1 year and another five were diagnosed with HIV after prison entry. Of prisoner participants who reported their likely mode of infection, the majority reported engaging in unprotected heterosexual intercourse prior to their imprisonment. Most (five) reported one or more years’ experience of living with HIV (with an overall median experience of 2.8 years) and using ART.

All prison health care staff and ART service providers had tertiary qualifications in health care. The prison health staff had nine or more years’ experience of working within the prison health care system, whereas the ART service providers had over 6 months’ experience of providing ART services. Prison officers, on the other hand, had two or more years’ experience of managing inmates’ visits to external health care facilities to access HIV care. Prison and health administrators were also experienced officials who had been managing and providing technical and material support for the prison health care system for four or more years.

While prisoners discussed best practices and challenges they experienced during initiating ART from structural, sociocultural and personal perspectives, service provider participants added diverse points of view to prisoners’ perspectives and described the pros and cons of the existing HIV care provision strategy for incarcerated people. Accordingly, we identified four themes as barriers to, and two themes as facilitators of, early ART initiation after analysing the in-depth interview data. Included under each theme are selected quotes that are representative reflections for the majority of participants. Pseudonyms (letters) are used instead of real names of the prisons, health care facilities and health departments in order to prevent potential identification of persons providing the information.

### Barriers to early ART initiation

#### Lack of access to HIV testing

The prison systems lacked testing facilities which would support early identification of HIV infected prisoners and linkage to care. New cases were detected only passively through ad hoc campaigns undertaken by external agencies, and when inmates requested testing due to severe sickness, suggesting an advancement of the infection. One prisoner reported:

*“My weight had been reduced severely but unfortunately I hadn’t realised that. I hadn’t known but something started to appear on my thigh [Showing his thigh]; um----something like weight loss, and it had just started making me dizzy when I had walked for a while, and tingling on the endings, then I was told I had the virus after being tested. They called me to the clinic, there is a clinic if you have seen it, and they called me there and gave me a piece of advice and took me to the hospital.” (Male prisoner, age: 30’s-40’s; Prison ‘B’).*

Other prisoners also reported having waited until an external agency came to the prison and performed testing, although they had noticed some possible signs of the infection and so wanted to have the diagnosis:

*“I got tested when [external] health professionals undertook a testing campaign. I was so sick long before I was aware of my status. There were times when testing campaigns were undertaken. A lot of inmates still want to have the test but they [prison health staff] often say ‘We don’t have test kits’.” (Female prisoner, age: 50’s-60’s; Prison ‘B’).*

Service provider participants supported the reports of inmates that there were inconsistencies in HIV testing services. This meant that inmates often undertook delayed testing at external health care facilities despite exhibiting symptoms suggestive of infection. Alternatively, they could incidentally be diagnosed through ad hoc testing campaigns undertaken by external agencies. A prison officer acting as a treatment facilitator described:

*“-------at least when they [prisoners] repeatedly come to our clinic with the same case, we take them straight to the hospital when their condition remains unimproved. No one has been dispatched from here to begin treatment there. But once, people from the Region [a health agency from the Regional State] took their blood for HIV testing and sent back the result via the Post Office.” (Male prison officer, age: 30’s-40’s; Prison ‘B’).*

Health agencies lacked faith in prison health care staff to undertake HIV testing despite the fact that they had been trained, and so denied test kits. Thus, HIV diagnostic services were external to the prison health care system and provided exclusively by public health care facilities. This represented a missed opportunity for prison health care staff to offer testing services and utilise the trust they had built with inmates through regular contacts, to perform the task. A prison nurse reported the following:

*“---- we had a training on the new kits because you have to have training whenever a new kit is launched, but they [health agents] were not happy to provide us the new kits assuming that we hadn’t had the training. They said, ‘We will do it ourselves.’ I don’t mean they shouldn’t perform testing but it would be better if it was performed by the prison itself because it is with us whom the prisoners make contact with, every day; it is us whom they trust more. It’s better when a person gets served by the professional whom he trusts more.” (Female prison nurse, age: 20’s-30’s; Prison ‘C’).*

Another prison nurse regretted that she was not able to undertake entry HIV testing for incoming prisoners due to an insufficiency of test kits, despite the fact that the majority of prisoners were eager to have the test:

*“-------It should have been [regarding offering prisoners voluntary HIV testing at entry], but we didn’t do that. By the way, all prisoners are voluntary to have HIV test, I can say. Many of them ask for testing when they come to the OPD (Outpatient Department). Seventy-five percent of them are very willing but I don’t have test kits, I am short of test kits. I just tell them, ‘Remind me when kits are available!’” (Female prison nurse, age: 30’s-40’s; Prison ‘B’).*

Prison officials confirmed a poor level of diagnostic services available in prisons and explained the process of testing prisoners for HIV. According to these participants, HIV testing occurred only rarely and usually in conjunction with international events such as on “World’s AIDS Day” or when external agencies carried out testing campaigns. A prison administrator elaborated on what he observed at his prison regarding HIV testing activities:

*“Mostly--- is it on the 22*^*nd*^ *of November? [referring to World’s AIDS Day]; it occurs on the 22*^*nd*^ *of November, and when there is a request by the Regional [Prison] Commission for an overall testing [program] including the staff, and umm-----in collaboration with the Zonal Health Department; they support us test kits.” (Male prison administrator, age: 40’s-50’s; Prison ‘C’).*

ART service providers and health agents also described that HIV diagnoses were made external to the prison health care system. They reported that the majority were conducted through incidental campaigns organised by outside agencies in the general community which gave no guarantee that inmates who were about to be released could access. Prison health care staff played little or no role on this, apart from referring prisoners to outside health care facilities when their health worsened. One ART service provider stated:

*“It is not actually the health professionals there [at the prison] who undertake testing and link positive cases, rather it is often through campaigns carried out by the City Health Unit, the Hospital or partners.” (Female ART service provider, age: 30’s-40’s; Health Facility ‘A’).*

Health agents felt a responsibility for making sure that every prisoner who volunteered for HIV testing received an opportunity ahead of his/her release. However, they were uncertain how effective they were in relation to the prison health care staff’s performance:

*“We do nothing! [regarding pre-release HIV testing]; We don’t know when he gets out. We never know; we never know [Laughs]! We have no any plan to test people who get out of prison, to be honest. We can’t work being there as a routine work. Umm-----they [prison health care staff] don’t ask us for help regarding educating people who come out of the prison.” (Male health agent, age: 40’s-50’s; Health Department ‘A’).*

In prison settings where onsite HIV testing rarely occurred, it was found to be only secondary to other health care activities. This impacted on the identification of HIV infected prisoners especially among new arrivals as the prison health care staff often gave a priority to other medical duties. A prison nurse described the situation and she recommended the presence of a separate office equipped with its own trained professionals to provide an effective diagnostic service:

*“-----they [new prison entrants] often come after 5pm from police stations; there is a high work load even if you want to perform [HIV]testing. It requires its own separate office and a professional who would undertake this work only. Many people may arrive at a time, more than a dozen of people! On one hand, it is not suitable to host them as there are people being served here [at the clinic]. I think it would be nice if there was a separate room and HIV trained professional who would perform this job; would be effective in identifying people who enter here every day.” (Female prison nurse, age: 30’s-40’s; Prison ‘B’).*

#### Inability of health staff to make timely care linkage

Prisoners who were able to be detected as HIV infected, either through testing campaigns or an opt-in diagnosis at a prison clinic, were not always provided their test results at the testing sites. They were often kept waiting for long periods of time. Prisoners described the circumstance that they were referred to a nearby public health care facility to learn about their HIV status, despite having the test in the prison:

*“The prison nurse made some part of the examination and told me that they had no kits to undertake a complete diagnosis so that he referred me to the nearby health centre. I had been told there that I was infected with HIV.” (Male prisoner, age: 30’s-40’s; Prison ‘D’).*

Another prisoner who tested positive in a campaign by external agencies said:

*“---------Then they [prison health care staff] called me to the clinic as it was a secret, they didn’t tell me anything except offering me an enveloped paper. Then they sent me there [to a hospital] and they [ART service providers at the hospital] had counselled me a lot and asked some questions.” (Male prisoner, age: 30’s-40’s; Prison ‘B’).*

ART service providers at external health care facilities described similar situations and found prisoners who were referred through such a system were confused about their test results after they realised that they were HIV infected. The service providers related the prison health staff’s inability to declare test results with an insufficiency of skills related to provision of appropriate counselling and referral services:

*“I have experienced something like this: He [a prisoner] was diagnosed there [at prison clinic] and we found him positive here and he said, ‘I was diagnosed there but I have not been told this!’ We just thought that it might be due to a counselling problem by the health staff and we counselled him and let him start the treatment.” (Male ART service provider, age: 40’s-50’s; Health Facility ‘B’).*

Another ART service provider gave an account of a prison health care staff member’s failure to offer proper post-test counselling adding their pejorative description of inmates’ being infected with HIV:

*“Umm---there was a prisoner who had newly been identified as HIV infected; I think there is some problem with the prison health staff; I don’t know whether it is due to a knowledge gap or being frightened; they don’t clearly let them know about their HIV status. Umm---they don’t tell them they have HIV virus rather they say like, ‘Your blood is turbid!’ We found the guy when he came to the Eye Clinic.” (Female ART service provider, age: 20’s-30’s; Health Facility ‘C’).*

A prison nurse acknowledged the problems associated with not letting inmates know their test results, and the fact that she never declared HIV test results to HIV positive prisoners; rather she referred them to nearby public health care facilities:

*“I mean, we don’t even let him [a prisoner] know his test result, although not recommended. We advise him, ‘I have tested you here but better you go to the health centre because they have more advanced testing equipment so that you can be more certain about your result!’ Then they test him again and offer him ART.” (Female prison nurse, age: 20’s-30’s; Prison ‘C’).*

ART service providers noticed significant delays even when such referrals were made that were not in accordance with standards of effectiveness and timing adhered to by other non-ART community health care facilities:

*“Among individuals who had been tested there [in prison], there are people who came after a month, two months, and even after four months. It is very difficult and requires a strong referral system. We have inter-ward and inter-facility linkage systems; other district health facilities do it in that way and we would have done the counselling here if they had told us the results even if they wouldn’t let the client know his result; if they let us know even using a piece of paper, or just sent it to us through the Post Office.” (Male ART service provider, age: 40’s-50’s; Health Facility ‘B’).*

Another ART service provider compared care linkage efforts made by the prison health care system and community non-ART sites, identifying a high likelihood of delays among incarcerated people even if the diagnosis was performed by similar health agencies:

*“The issue of care linkage is the usual complaint. Prison campaigns have been undertaken and positive cases were identified; but if it was in the community, there would have been a high chance of being immediately linked to care. For instance, if a positive case is found in a community campaign, one can easily bring him here, and health professionals can also easily bring them if found here in the hospital. However, the situation at the prison is really hard.” (Female ART service provider, age: 30’s-40’s; Health Facility ‘A’).*

A prison nurse also described the presence of considerable delays in care linkage while attempting to assemble patient inmates to send them to external health care facilities en masse:

*“If they [prisoners] are found to be positive today, I will call them today. However, the number of people matters when we send them to the hospital. If not urgent, I will suspend patients who have an appointment today for tomorrow to include them. If so, I’ll look at the appointment and say, ‘I’ll send you on this day!’ I say, ‘Stay ready!’ it won’t be longer than a maximum of a week.” (Female prison nurse, age: 30’s-40’s; Prison ‘B’).*

#### Uncooperative prison security system

Prison security’s denial of inmate requests for external health care facility visits played a role in causing delays in care linkage. It tended to discourage newly identified HIV-infected prisoners from pursuing ART initiation and even to deny that they were infected. One prisoner described how he noticed his friend dissuading himself from ART initiation because of the emotional trauma he experienced as a result of prison security’s procrastination about his health care facility visit:

*“One day, they [prison security] gave him [newly identified HIV infected inmate] an appointment and let him be back. He became very offended since then. ‘You didn’t take me out at my appointed time so I don’t want to go again!’ he refused. They had declined to take him to the health centre a couple of times due to a cloudy weather. He got frustrated because of this and he was even saying, ‘I don’t have the virus!’ [Laughs].” (Male prisoner, age: 40’s-50’s; Prison ‘B’).*

A health agent also reported a prison officers’ denial of external health care facility visits as a barrier to accessing care amongst HIV infected prisoners:

*“Sometimes these people [HIV-infected prisoners] may not come [to an external health care facility] by themselves because they have low access to outside environment. At times the prison officers refuse to bring them to the health facilities.” (Male health agent, age: 50’s-60’s; Health Department ‘C’).*

#### Loss of privacy regarding HIV status

Prisoners sometimes refused to be initiated on ART due to concerns about loss of privacy during procedures at external health care facility visits, as well as negative attitudes displayed by prison officers during the process. One prison nurse shared her experience in relation to this while assisting newly identified HIV infected inmates to start ART, proposing onsite ART services as an ideal approach to avoid such difficulties:

*“---------This was the main reason why the guy we talked about earlier refused to start treatment. He had been tested here and the prison officers tried to take him to the health centre. He replied, ‘I don’t want to go there!’ It is a very bureaucratic procedure. They should be tested and start their treatment here at the OPD (Outpatient Department). It reduces mistreatment for the prisoners.” (Female prison nurse, age: 20’s-30’s; Prison ‘C’).*

Another prison nurse described the occurrence of privacy loss during call-backs of HIV positive inmates to let the inmates know their test results, because of the involvement of a third party (prison officers and other prisoners):

*“------If so [referring to being tested positive], we’ll call [back] and let them [prisoners] know. But when they are called out alone, other inmates become suspicious. If you say to someone [a police officer], ‘Get a person with this number!’ he himself will be suspicious. There is something like, ‘He was called because he has the virus!’” (Female prison nurse, age: 30’s-40’s; Prison ‘B’).*

On some occasions, HIV positive prisoners were not directly informed about their test results, but prison officers were informed about the results prior to taking them to public health care facilities. On these occasions, prisoners were unaware of why they had been escorted to the external health care facility until informed by the ART service provider. One ART service provider described:

*“It was one of the guarding police who told me, ‘He [a prisoner] has tested positive [Whispering]!’: ‘He came here after being diagnosed there [at prison clinic]!’ The man didn’t know, but the guarding police knew. The prisoner says nothing. It’s just the person pulling him in and out.” (Male ART service provider, age: 40’s-50’s; Health Facility ‘B’).*

### Facilitators of early ART initiation

#### Peer education and support

Participants discussed the importance of peer education and support for having an early diagnosis and status disclosure to access care in the prison environment. Peer support of ILWH was identified as an essential source of information and a means through which the more experienced ILWH convince newly diagnosed inmates to start treatment. As a prisoner who had been using ART in prison for about 4 years said:

*“We are the ones to help them [HIV infected prisoners who refused to be initiated on ART]. If we seniors advise them, they will take it easy and start their medication. Otherwise, they fear to ask and may get worst.” (Male prisoner, age: 40’s-50’s; Prison ‘B’).*

Although ILWH highlighted the significance of sharing their experience of living with HIV and indicated their intention to perform the course of action, it appeared to be challenging for them to participate in peer education activities that were seldom held in the correctional facilities. They encountered an interference by people without HIV experience in the educational programs that was apparently unnoticed by prison officials. A prisoner discusses:

*“Yes, it [referring to World’s AIDS Day] is celebrated here once in a year, but when that occurs, it is mainly city gangsters who engage in the ceremonial activities. They just interfere in every activity, they know how to dance, how to talk, and then they will be paid! They are the ones who dance and teach, no one who lives with HIV has ever come in. There is no one to coordinate us. They [prison officials] still remain unresponsive.”*

*“I am willing personally [to share his life experience with others]. I don’t even teach at a ceremony, why not they print my name out in newspapers! I will teach them “Why and how it occurs!” But it was not given to me, it was given to the gangsters. They don’t know the extent that I know about the situation, it is just an intrusion.” (Male prisoner, age: 30’s-40’s; Prison ‘B’).*

A prison nurse who had previously run an HIV prevention office described the cessation of HIV education programs at her institution despite the commitment of ILWH to educate fellow inmates. She attributed blame for the interruption to a disregard for the program by prison administration and health agencies:

*“There had been tea-coffee programs. We used to be provided with two-percent of the total institutional budget for a monthly tea-coffee program; an exciting program! Umm----the Zonal Mainstreaming Officer used to attend the program [I don’t know who is in charge of the Office currently; might have been changed], and provide us with brochures, music CDs, and teaching leaflets. The prisoners [living with HIV] also used to write a poem, and it was really a vibrant ceremony. It has been interrupted now, otherwise it was an exciting activity.” (Female prison nurse, age: 20’s-30’s; Prison ‘C’).*

Prison health care staff did not always feel it was their responsibility to undertake HIV education programs:

*“It’s just like you sit in the clinic and do the work you are supposed to do, but there is nothing else you can feel as a responsibility [regarding HIV education]. We didn’t have love and unity. There was no thought to each other within the team rather fault finding. Then you would go out having done your work to which you are accountable for. That has created the gap.” (Female prison nurse, age: 30’s-40’s; Prison ‘B’).*

Both prison and health administrators blamed supporting agencies and the government for a reduction in allocation of resources related to HIV prevention and control activities. It was assumed that the infection had meaningfully declined in the community, which eventually evoked restriction of funds by donor agencies. Having announced the decline of the infection in the community, the government did not appear to have the capacity to implement the programs on its own, which had previously been operated by donor agencies in the main. However, the infection continued to spread at an epidemic level, particularly among the most at risk populations. A health agent described the active HIV prevention and control programs that previously existed at the Zonal level, and the anticipated risks that health agencies would likely face in attempting to achieve the goal of ending the AIDS epidemic by 2030 if funds by donor agencies remained restricted:

*“There is no one to be asked [for funds] like before. Everyone is short of funds. HIV has been assumed to decline but it has not actually; it has been disseminating like a wild fire [Laughs]! A lot had been done at schools, districts and neighbourhood level including prisons. Following these all efforts, the Ethiopian government declared that HIV had been reduced by 70%; [consequently] the budget has declined considerably since then. The Global Fund and supports of USAID have been shifted to other issues leaving the country to deal HIV issues on its own.*

*While the national government announced HIV had been reduced, be it for political purpose or not, but it still remains at the epidemic stage. According to the international definition, the prevalence of more than 1% in the general population is considered as an epidemic. But there are cities in our country with a prevalence of more than 5%. Hosanna [the City where his office located] itself has documented over 2%, the land where HIV was assumed to be absent. In this sense, the government and the people got distracted. It has set to do that again but it is not going to be effective by the government’s only capacity. Although the government is saying, ‘HIV will be stopped by 2030’ it’s getting harder.” (Male health agent, age: 40’s-50’s; Health Department ‘A’).*

A prison administrator added:

*“Generally, as there has been a decline in HIV related activities, particularly in relation to the recent slogan, ‘Our achievements on decline’; there must be awareness creating work to enhance this through umm----drama, umm------conversations and other means to create knowledge amongst high risk groups such as drivers, soldiers, and others groups like prisoners; both men and women need to know that it [HIV infection] occurs due to lack of precautions to protect oneself.” (Male prison administrator, age: 40’s-50’s; Prison ‘C’).*

In addition to the decline in the emphasis on HIV-related issues at national level, prison administrations and health agencies failed to work collaboratively or demonstrate appropriate understanding that prisoners are among the most at risk populations for HIV transmission:

*“Not that much in this regard [participation in the development and implementation of HIV related plans at the Zonal level]; they don’t invite us in what [HIV related plans] they have developed” (Male prison administrator, age: 40’s-50’s; Prison ‘C’).*

The same prison official discussed the apparent gap in HIV education activities existing between his institution and health agencies, and his perception that it was partly attributable to the prison administration’s lack of mandate to make direct contact with health agencies. He proposed he was forced by the circumstance from involvement in the implementation of HIV-related programs at Zonal level:

*“As I said it before, we have no a mandate to directly attract NGOs (non-governmental organisations) to our institution or contact Regional Health Bureau because we are responsible to the Regional Prison Commission. It should be decentralised but still they are the ones who contact NGOs to give us holistic trainings wherever they come from. For instance, you came here after having reported to the Regional Prison Commission! So?” (Male prison administrator, age: 40’s-50’s; Prison ‘C’).*

#### Imprisonment as an opportunity for early ART initiation

Service provider participants discussed the prospects that incarceration could offer for early treatment of HIV infection. A health agent viewed incarceration as an opportunity to identify and initiate ART for HIV-infected individuals who otherwise might have been difficult to reach:

*“For instance, sometimes you may not find HIV-infected people at health facilities but you may find them at prisons. They may refuse to start ART as they might have tested [positive] at private clinics. Thus, prisons provide a good opportunity to capture such cases which would benefit the patient as well as the community at large.” (Male health agent, age: 50’s-60’s; Health Department ‘C’).*

One prison nurse appreciated the importance of vicarious experiences of the valuable outcomes of ART that a prison environment offered ILWH. She provided more weight to positive and negative outcomes in fellow ILWH in changing their behaviour than education provided by health care providers. In her perception, it helped ILWH understand the health benefits of ART and decide to initiate:

*“It is not because we have educated them [prisoners] correctly or advised them, ‘the medication does this in your body, it reduces viral load, it boosts your immunity’, but they learn from the people inside. For example, I’ve experienced this: many had just been so drained and their body got back to normal after they had started the medication. Many others have learned from this. They believe that ‘Mr ‘X’ was like this so nothing happens to me!’” (Female prison nurse, age: 30’s-40’s; Prison ‘B’).*

One female prisoner reported having HIV diagnosis after prison entry, which she was incapable of performing before imprisonment even if she was suspicious about being infected due to her partner’s death related to HIV:

*“I was not diagnosed even after his [her husband’s] death, when I was outside prison. Then this crime was committed, came into prison; it was after my prison entry that I got diagnosed.” (Female prisoner, age: 50’s-60’s; Prison ‘B’).*

## Discussion

In this study, we identified and explored various barriers to and facilitators of early ART initiation amongst prisoners in South Ethiopia. Among the barriers identified, there was a lack of enduring strategies and resources that ensure access to HIV testing which led prisoners to commence ART only at advanced stages of their infection. Ensuring access to voluntary and confidential HIV testing for prisoners is an essential component of efforts to reach universal access to HIV prevention, treatment and care [55]. This enables the HIV key population to undergo early diagnosis, which is an important prerequisite for timely ART initiation [17], improved treatment outcomes [18, 19] and reduced risk of infection for others [20, 21].

Participants reported that HIV testing was usually only available upon the prisoners’ request (also known as opt-in or risk-based approach). Such approaches are less effective in correctional settings resulting in delayed diagnosis and linkage to care, and provide a little opportunity to reach prisoners before they return to the community [56–58]. In South Ethiopia, the majority (86%) of prisoners return to the community within less than 2 years of imprisonment [51], which could make an effective implementation of opt-in approach challenging. A number of other institutional and social factors also affect prisoners’ ability to request testing. As stated by some prisoner participants, they often faced refusal from health care providers and prison officers when they tried to seek testing. In addition, due to widespread social stigma and discrimination against HIV both in the community and prison settings, individuals who suspect they may be infected with HIV are generally less likely to request testing [55, 59–62].

In contrast, several intervention studies suggest the effectiveness and feasibility of implementing VCT in prison settings in high- and low-income countries. For example, opt-out based provider initiated counselling and testing (PICT) approach has been associated with higher testing rates in many prisons when integrated into routine prison health care systems [23, 24, 58, 63]. This approach is also known to increase rates of linkage to care and ART initiation especially in HIV infected prison entrants [23]. Adaptation of the Seek, Test, and Treat (STT) strategy [64] which involves identification and offering of ART to all HIV-infected individuals, may further ensure universal access to testing and treatment services for all infected prisoners.

Insufficient training for prison health staff on pre- and post-test counselling resulted in delay in the feedback of test results and linkage of HIV-infected prisoners to external ART sites. ART service providers confirmed greater delays in care linkage for prisoners relative to people who were tested at non-ART sites in the community. Training of prison health staff is an essential step in scaling up access to HIV testing, care and treatment for prisoners [55]. This is supported by public health recommendations in international guidelines in relation to training and reassigning tasks to lower level health care workers to enhance testing coverages in HIV key populations [17, 65]. Such approaches are also found to be efficient and effective in increasing testing rates in prisons in low-income countries, particularly when coupled with peer education programs [63].

Given the congregated living conditions in the prisons (up to 150 inmates accommodated together in a cell measuring 100m^2^) [51], patient privacy was lost on various occasions. Loss of privacy occurred while declaring test results and transportation to external health care facility to access care, which led prisoners to refuse treatment. Prison officers’ negative perceptions towards HIV infected prisoners during the process of external health care facility visits also contributed to loss of privacy and delays in care linkage. That they allowed third parties to be privy to prisoners’ HIV status through openly feeding back test results, indicates that prison health care staff lacked skills in ensuring patient privacy and confidentiality.

Studies in low-income countries also reported accessing HIV care from external health care facilities and the associated prison officers’ uncooperativeness as major causes for loss of privacy and missing clinic appointments amongst HIV infected prisoners [66]. Conversely, higher rates of linkage to care and ART initiation were achieved through an onsite ART approach in many prisons [22, 23, 67], which was also supported by most service providers in this study. There are no legal grounds to ensure medical privacy for prisoners in Ethiopia, and it seems to be a difficult task given the circumstances related to accessing care in groups and a congregated living environment. However, provision of appropriate counselling for prisoners during diagnosis and reducing social stigma by creating awareness about HIV amongst members of the prison community may encourage consented discloser. Training in patient privacy and confidentiality for prison staff who are involved in the provision of HIV care could improve undisclosed inmates trust and, consequently, uptake of ART [55].

The majority of participants described the importance of peer education and support programs for early diagnosis and treatment of HIV infection in prisoners. Such programs not only offered ILWH an opportunity to share each other’s experiences of living with HIV in a prison environment, but also served as a route to influence those who lacked motivation to commence treatment. At the time of the interviews, however, there had been a gradual decline in HIV education and social support initiatives in the prisons. This was explained as being due to a lack of resources and administrative support, and poor collaboration between prison authorities and external health agencies.

Social support and education initiatives involving peers are highly recommended in correctional settings to enhance HIV testing and to access care [14, 68]. Prisons represent important organisational contexts to identify HIV infected individuals and implement peer education programs to enhance early treatment initiation [69]. Prison HIV prevention and control programs should be an integral part of community information, education and communication programs, and prison authorities should establish strong linkages with these community-based health agencies, in addition to developing plans to assure appropriate resource for supporting well organised peer support and education programs [68].

This study explored important factors influencing early ART initiation among prisoners, but does have some notable limitations. Most prisoner participants had been using ART for many years and this might have affected their recall in relation to their experiences when initiating ART. At the time of the study, there were no identified HIV-infected prisoners who had not already initiated on ART, which may have impacted on identification of all barriers to treatment in this setting and represents an area for future studies. While a number of crucial concepts emerged through consultation with service providers regarding the effectiveness of the existing HIV care provision strategy, incorporating higher level health agencies (e.g. representatives from the Regional Health Bureau and Ministry of Health) might have deepened our understanding of the policy perspectives of HIV care in the prison settings. Although most structural factors identified and explored in this study have been interpreted as strongly influencing prisoners infected with HIV, a quantitative survey with a large sample size is now required, based on these qualitative results, to provide a representative sample regarding the determinants of early ART initiation.

## Conclusions

Participants in this study discussed both barriers to, and facilitators of, early ART initiation in prisoners. The barriers include a lack of access to HIV testing services, poor linkage to care due to insufficient health staff training, uncooperative prison security systems and loss of privacy regarding HIV status. Insufficient health staff training and uncooperative prison security both contributed to loss of patient privacy, which resulted in treatment refusal. Although most participants described the importance of peer education and support for enhancing HIV testing and treatment programs amongst prisoners, there had been a decline in such interventions in the correctional facilities due to lack of resources and administrative support. Service providers identified opportunities offered by the prison environment for identification and treatment of HIV-infected individuals and implementation of peer education programs. Timely initiation of ART amongst inmates is likely to be enhanced through interventions that ensure access to voluntary and confidential (opt-out based) HIV testing and care, peer education and support programs, and training of health care providers and other prison staff who are involved in HIV care provision.

## Supplementary Information

**Additional file 1.** Prisoners and service providers interview guide. An interview guide for prisoners, prison officers, prison health staff, ART service providers, prison and health administrators.

## Data Availability

The datasets used and/or analysed during the current study are available from the corresponding author on reasonable request.

## Abbreviations

AIDS: Acquired Immunodeficiency Syndrome
ART: Antiretroviral therapy
HIV: Human Immunodeficiency Virus
CD4: Cluster of differentiation-4
ILWH: Inmates living with HIV
PICT: Provider initiated counselling and testing
RHB: Regional Health Bureau
SBREC: Social and Behavioural Research Ethics Committee
SNNPR: Southern Nations, Nationalities and People’s Region
SSA: Sub-Saharan Africa
VCT: Voluntary counselling and testing
WHO: World Health Organization
