# Supplementary material for: Various structural factors influenced early antiretroviral therapy initiation amongst HIV infected prisoners: a qualitative exploration in South Ethiopia
Source: BMC Public Health. 2021 Jul 28;21:1463. doi: 10.1186/s12889-021-11499-w (PMC8317278; doi:10.1186/s12889-021-11499-w)
Supplement: Supplementary file 1 — Additional file 1. Prisoners and service providers interview guide. An interview guide for prisoners, prison officers, prison health staff, ART service providers, prison and health administrators. [file 12889_2021_11499_MOESM1_ESM.docx]

| **Prisoners and service providers interview guide** | | | |
| --- | --- | --- | --- |
| **(A) Prisoners interview** | | | |
| 1. | Welcome and brief introduction about the study |  |  |
| 2. | Personal information: age, sex, educational and marital status |  |  |
| 3. | Where you were first diagnosed for HIV? Would you describe how you got diagnosed? (Probe: steps a participant followed to get HIV diagnosis in the prison) | -------------- |  |
| 4. | How long did you wait to get HIV treatment? Why did you stay for this amount of time without treatment? (Probe: personal and institutional barriers to access ART while starting treatment) | -------------- |  |
| 5. | What is the level of importance that you attach to ART for your health? (Probe: ART benefits, risks of treatment interruption ) |  |  |
| 6. | Describe the way you are receiving ART drugs in the prison? Are you satisfied with it? If not why? (Probe: processes and length of time required to access drugs, distance between clinic and prison/cell, means of transportation, people involved) | -------------- |  |
| 7. | Describe any challenges you might be facing in accessing ART drugs and why the challenges exist? (Probe: challenge due to lack of cooperation, transportation, distance, lack of drugs, lack of privacy and associated stigma/discrimination) | -------------- |  |
| 8. | Have you ever faced HIV related stigma and discrimination in the prison? If yes, how would you describe it? (Probe: particularly associated with accessing and taking drugs) | ------------- |  |
| 9. | Describe the level of privacy in taking ART drugs in the prison and the amount of confidentiality offered by health staff and officers? | -------------- |  |
| 10. | In general, how would you describe the prison officer’s attitude towards HIV positive inmates? (Probe: related to routine treatment compared to HIV negative inmates) | -------------- |  |
| 11. | How would you describe the ART staff’s attitude towards HIV positive inmates? (Probe: compared to community based HIV positive people) | -------------- |  |
| 12. | What support do you get from prison health staff? How do you describe it? (Probe: in terms of HIV diagnosis, treatment initiation, accessing ART and counselling/support to sustain adherence (schedule)) | ------------- |  |
| 13. | How would you describe care and support provided by prison officers to HIV infected inmates to get HIV care? (Probe: when a patient seeks support and during health facility visit) | -------------- |  |
| 14. | How would you describe the quality of care you receive in public health facility in terms of ART drug availability, time, respect, privacy and confidentiality? |  |  |
| 15. | Do you have any suggestions about what could be done to improve the ART service in prison? | -------------- |  |
| **(B) Prison officers interview** | | | |
| 1. | Welcome and brief introduction about the study |  |  |
| 2. | Could you describe your role in HIV care in the prison (if any)? |  |  |
| 3. | Could you describe any challenges that might exist for HIV positive inmates in accessing ART drugs and why they exist? | -------------- |  |
| 4. | How would you describe the prisoners’ interest to attend clinic appointments and collect medications? | -------------- |  |
| 5. | If any, what support are you providing for HIV positive prisoners with and without ART and how? | -------------- |  |
| 6. | Is there anything you would suggest that could be done to improve ART service in the prison? | ------------- |  |
| **(C) Prison health staff interview** | | | |
| 1. | Welcome and brief introduction about the study |  |  |
| 2. | Would you describe your role in HIV care in the prison (if any)? | ------------- |  |
| 3. | How long have you been doing this job? |  |  |
| 4. | What training have you received regarding HIV care? |  |  |
| 5. | How would you describe HIV care in the prison in terms of diagnosis, treatment initiation and adherence support? | ------------- |  |
| 6. | How are TB infected inmates being identified in the prison? | -------------- |  |
| 7. | Would you describe what challenges exist for HIV positive inmates in accessing ART drugs, receiving CD4 and viral load tests? If there is any, why do you think they exist? | -------------- |  |
| 8. | How would you see prisoner’s knowledge about and trust in ART? Is there any action being taken to scale up inmates knowledge and trust in ART? | -------------- |  |
| 9. | How would you see privacy issues for HIV positive inmates in taking ART drugs and protection of confidentiality? | -------------- |  |
| 10. | How would you describe HIV related stigma and discrimination in the prison? | ------------- |  |
| 11. | What kind of support do you provide for HIV positive prisoners and how? If so, is there a standard document for that?  a. not started ART  b. on ART | -------------- |  |
| 12. | Would you describe what is being done to prevent HIV in the prison? | ------------- |  |
| 13. | Would you describe what is being done to ensure continuity of HIV care including ART for prisoners arriving in, and leaving prison? | ------------ |  |
| 14. | How would you describe training level of prison officers about the importance of continuity of HIV treatment? |  |  |
| 15. | What would you suggest to be done in order to improve ART service in the prison? | ----------- |  |
| **(D) ART service providers interview** | | | |
| 1. | Welcome and brief introduction about the study |  |  |
| 2. | Would you describe your role in HIV care? | ------------ |  |
| 3. | How long have you been doing this job? | ------------ |  |
| 4. | What training have you received regarding HIV care? | ------------ |  |
| 5. | Are ART drugs always available in the health facility? (If not, how often and why?) |  |  |
| 6. | How reliable are the laboratory services in the health facility? Are CD4 and viral load tests done on time? Are results delivered on time? (If not, please explain why?) |  |  |
| 7. | How do you identify HIV positive prisoners? (Probe: coordination between prison health staff and ART staff) | --------- |  |
| 8. | How do you link HIV diagnosed prisoners in to care? | ------------ |  |
| 9. | What kind of support do you provide for HIV positive prisoners and how? If so, is there a standard document for that?  a. not started ART  b. on ART | ------------ |  |
| 10. | How would you evaluate HIV positive prisoners’ HIV care utilization compared to community people in terms of attending appointments, treatment initiation, drug pickups and adherence compared to people from the community? | --------------------------- |  |
| 11. | What are the main challenges you face in supporting HIV positive prisoners to get HIV care compared to people from the community? | ----------------------- |  |
| 12. | How would you describe prisoner’s interest to visit a public health facility? (Probe: any frustration, complain, or treatment refusal) | ------------------------ |  |
| 13. | How would you see prisoner’s knowledge about and trust in ART compared to people from the community? | ------------ |  |
| 14. | What would you suggest to be done in order to improve ART service in the prison? | ------------ |  |
| **(E) Prison and health administrators interview** | | | |
| 1. | Welcome and brief introduction about the study |  |  |
| 2. | Would you describe your role in HIV care in prison/s? | ---------------------- |  |
| 3. | Is the prison authority taking part in the HIV/AIDS coordination activities in the zone? If so, how? Can you show evidence/document for that? | --------------------------- |  |
| 4. | Is the prison HIV care issue part of the HIV action framework and monitoring and evaluation system at zonal-level? If so, how? | --------------------------- |  |
| 5. | Does the prison authority involve in all aspects of treatment scale-up, from applications for funding to development, implementation, and monitoring and evaluation of treatment roll-out plans? If so, how? | --------------------------- |  |
| 6. | What policies, guidelines and systems are available specifying that people with HIV or AIDS are allowed to keep their HIV medication upon them, or are to be provided with their medication, upon arrest and incarceration and at any time they are transferred within the system or to court hearings? | --------------------------- |  |
| 7. | How would you describe training level of prison officers about the importance of continuity of HIV treatment? | --------------------------- |  |
| 8. | Does the prison have any partnerships with health clinics, hospitals, NGOs, universities and civil society organizations to provide health care and other services for prisoners? If so, please explain how? | --------------------------- |  |
| 9. | How would you describe prison health care staff’s training level in the comprehensive management of HIV and AIDS, including the provision of antiretroviral therapy? | --------------------------- |  |
| 10. | How would you evaluate the existing strategy of antiretroviral therapy service in the prison? (Probe: HIV diagnosis, treatment initiation, access to ART and adherence support) | --------------------------- |  |
| 11. | What would you suggest to be done in order to improve ART service in the prison/s? | --------------------------- |  |
